# Tri-State Meeting, June, 1898

**Published:** 1899-02

**Authors:** C. S. Case

**Affiliations:** Chicago


### Proceedings.

### Tri-State Meeting, June, 1898.

[*Continued from December No., Page* 591.]

### "DEVELOPMENT OF FACIAL CONTOURS BY MOVING THE TEETH."—ABSTRACT.

BY DR. C. S. CASE, CHICAGO.

Wednesday afternoon. Meeting called to order by Dr. S. B. Hartman, Ft. Wayne, Ind.

Mr. President, Ladies and Gentlemen: I would like to say before commencing to read my paper that I have presented a number of papers at different dental societies upon a subject similar to the one which I am to present to-day, but yet these were entirely different; I do not want you to think I am going to give you a rehash of the same old thing. This is the first time before any dental society that I have attempted to present this subject in a systematic manner, taking up the different deformities of the face caused by irregularity of the teeth, with an attempt to describe to you systematically how these several deformities shall be corrected. It might seem to many who come to these lectures and see the display of models, that it was a sort of grand-stand display to create a sort of admiration on your part or otherwise; in fact I have heard that such expressions have been made, but nothing could be further from the truth. The fact is, in my estimation, this work of facial development will be found to lay at the very foundation of everything in orthodontia, "the science of facial contour by applying force to the teeth."

Although it is a fact that every individual is correct in structure and form by the laws of inheritance, we are nevertheless constantly brought face to face with differences of the purely local organization that have been developed during immaturity, resulting from outside forces. Under the head of local causes may be mentioned first the almost universal habit in the use of one side in grinding, producing a marked lack of symmetry in the form of the physiognomy. Often the entire lower portion of face is thrown out of alignment principally because the lower jaw has been, during its growth, forced to one side. It is also common to see the nose bent to one side. In fact, if it were possible for us to compare by exact measurements one side of face with the other we would be quite surprised at the difference which exists in almost every face we meet. I do not claim that these differences are always produced from local causes, but if mothers were informed as to the influence they exert upon the future physiognomies of their children, and also the influence they might exert by skillful manipulative movements, the physical forms of future men and women would be far more perfect.

Mal-occlusion is another local cause which sometimes results in changing shape of jaw, external contour and expression of the physiognomy. It is reasonable to assume that local causes exerting a slight, constant force upon immature muscles and their bones, during the early stages of growth, have somewhat the same effect which is known to be possible later, by artificial force. For example: The models which accompany the casts of the faces, it will be found, are numbered with the same as that attached to face; so by looking at any one of these at any time you can, by casting your eye upon the number, see the model of the face of the same cast, because I have models of teeth of every one of the cases which I present before the commencement of treatment and after the final finishing of it.

Patient aged thirteen years. When presented the upper incisors were posterior to a normal position and so badly in lack of occlusion that the upper crowns were nearly hidden behind the lowers. With the exception of upper cuspids, which were forced slightly out of alignment, all the other teeth were in their proper places and occlusion. The posterior position was not due to the lingual inclinations of their crowns, but the reclusion extended to the roots and entire inter-maxillary portion of jaw having a decided impression on overlying features, with the effect of a protruded lower jaw.

History of case: The lower incisors erupted much earlier than upper and with the short bite occlusion; as soon as the upper incisors began to erupt they became interlaced with the lowers. Roots and surrounding process were in an immature position as the course continued.

By examining the models of this patient you will see the one at the beginning represents all that portion of space lying between the cuspids in a position very much posterior to the normal one, whereas in the final model all that arrangement is carried forward and teeth are still in an upright position. As the cuspids and bicuspids come into place the lateral portion of the jaw was allowed to develop in harmony with the lateral growth of the mouth; these cuspids and bicuspids will be found in their proper relative position as regards the lower ones. The force was applied to upper incisors alone. In less than six months the entire case was completed with the perfect correction of a very unhappy facial deformity.

Another case: Caused by extraction of laterals to permit cuspids to erupt. In presenting these models I wish to say I did not expect to bring them before this society, because I thought it was not complete. But with a new appliance that we have recently invented for retaining teeth that have been moved bodily forward, which acts as retaining appliance also as contouring appliance, I concluded to remove the contouring appliance she had worn, and the models which I have here show the position of the teeth before treatment in the mouth of a young lady twenty-four years of age. I did not expect to make quite as successful a conclusion of the operation as I have and I did not take a face model before I started, but I have appended to this a photograph that she gave me a few days ago which was taken before she commenced the operation and before she thought of having anything done. It will give you an idea of her appearance.

Another case, very similar to the above, was caused by extraction of laterals to permit the cuspids to erupt. A dentist extracted those lateral incisors just to allow the cuspids to come in, and by so doing prevented the proper development of all the anterior portion of maxillary arch.

If I can do anything in the line of getting the dentists to use their eyes a little and put them on to the method of treatment their patients shall receive, it will be just the work I hope to accomplish.

Other conditions, such as characteristic deformities caused by mouth-breathing and thumb-sucking which are so well known and recognized that it will require no mention of in this paper. I could give many models illustrating this.

My object in presenting these ideas is to show how the bones of the human body, and especially those of the framework of the physiognomy, are frequently changed by natural local causes from the forms which nature intended them.

If such things are accomplished by natural force, we have every reason to believe by intelligent application of artificial force during the period of immaturity we can æsthetically protect the features over all that area which can be influenced by movement of teeth and immediate surrounding bone, as I have explained elsewhere, by applying force directly to bone itself. The teeth imbedded in the alveolar process, that in turn is firmly united to the true bone, when attached to the regulating appliance, may be considered as an integral part of it firmly and directly attached to that part of the bone we desire to move. This force being applied, united to many teeth side by side, the surrounding bone is carried bodily in the direction of the force; not by fracture of substance but by elongation of cellular structure, bodily adapting itself to a new form, in which position it is brought to equilibrium by nature. It may be required to be held in that position for many months.

In contemplating the treatment of dental irregularity, a careful study of the physiognomy in different attitudes of expression should be made, with a view of determining the relative position of face and contour. The portion of the human face which is possible to be changed by a dental regulating appliance may be said to lie between two vertical lines which may be said to arise at a point below the bridge of the nose and go down the side of the nose, then laterally to encircle a part of the cheek and then down to the angles of chin. It is in this place which is produced marked changes in the contour, with characteristic physical differences. It may be called the changeable area. For convenience of ready reference it is divided into four segments: No. 1. The end of nose and upper portion of upper lip, including the naso-labial depression. No. 2. The lower portion of upper lip. No. 3. The lower lip to the commencement of chin. No. 4. The chin.

In a preliminary examination of the physiognomy from a purely æsthetical standpoint, with a view of correcting the features by applying force to the teeth, there are certain things to be noted. There are two classes: 1st. Those which lie in the unchangeable area, as the forehead, bridge of nose and malar prominence. 2d. Those in the changeable area. The four segments in the changeable area shown in Figure 5 are changeable in their relation to each other and also in their individual relations to the features in the unchangeable area. For instance: It is possible to protrude or retrude the upper portion of upper lip without changing the lower portion of upper lip in its relation to other parts. The same is true of the other segments, in fact a retrusion of the segment and the protrusion of the first may be accomplished at the same time, and *vice versa*. I will show from models cases of these two changes. In the casual examination of profiles we frequently observe a lack of perfect equanimity in the positions of the chin, the lower jaw is frequently protruded or retruded so as to lower æsthetic condition of the physiognomy, and yet if these were examined in the majority of cases we would find the lower jaw was in perfect harmony with the unchangeable area, and that the appearance of the mal-position was a fact due wholly to the protrusion or retrusion of the upper jaw and teeth. In other words, it is a common error to imagine the chin imperfectly posed because it is not in harmonious relation with the other features of the changeable area instead of comparing it to the unchangeable features of the physiognomy. (Examples of profiles cut from pasteboard). In examining the physiognomy of patient the head should be in an upright position on the line of the observer and the face studied from different angles. The most important thing to determine is the relative position of chin with the forehead, molar prominence and bridge of nose, the features in the unchangeable area. If its position is harmonious with these features and upper lip is well posed the operation of facial contouring should be performed upon the upper jaw and teeth, for, as has been shown, such an appearance in the first and second areas of the changeable area will cause an appearance of retrusion of the chin and lower jaw, and *vice versa*.

### UPPER DENTAL AND MAXILLARY PROTRUSIONS.

There are five distinct characteristics of this deformity—abnormal upper protrusions, divided according to the treatment which the physiognomy demands to bring about a most perfect æsthetic effect. The relative appearance of a protrusion is largely due to the retrusion of the lower jaw. Instances arise if it were possible and practicable to permanently protrude the entire lower jaw, the width of the bicuspid, or perform the operation of "jumping the bite," the results would be far more perfect than it would be to accomplish the work without it. I have never systematically tried to accomplish this irregularity. While there are successful operators who seem to think that the operation of "jumping the bite" is demanded in all cases of upper protrusions, I must say I believe these cases to be rare, and nine out of ten which have been attempted have failed. A far more satisfactory result could have been accomplished by bodily retruding the upper teeth and alveolar ridge. In other words, the chin in its relation to the unchangeable area will be found rarely ever, far from its æsthetic conditions. The reason is that it is compared with the central features of the physiognomy.

Figure 7. Ordinary class of abnormal upper protrusions. If the operation of "jumping the bite" be performed in this case there would no doubt be an improvement of the appearance of the physiognomy. The process involved in the treatment of these deformities may be illustrated as follows. It is usually done by bringing the lower jaw in, but this is no good. I have drawn this chart with a view of representing the ordinary upper protrusions. If now we protrude the lower jaw we find an improvement in the appearance of the physiognomy, because we are comparing the lower immediately with the upper, but the improvement is not as great as it would be if, instead of protruding the lower, we retrude the upper. A black card placed over these shows what a small change has been made.

Figure 9. Typical case of abnormal protrusion of upper jaw.

Figure 10 shows the improved effect by jumping the bite in bringing segments three and four into more perfect harmony with one and two, yet this is not to be compared to that symmetrical contour shown in Figure 11, where chin and lower lip remain in their normal position while the upper lip and nose are thrown back. In cases of protrusion by applying a retarding force especially constructed to roots and crowns of anterior teeth of the front alveolar process will be forced back, allowing the lower lip to fall into graceful repose. If due to a labial inclination of large, crowded teeth with no marked protrusion in segment No. 1, the application of force is put on crowns of these teeth in such direction as to overcome the mal-occlusion.

Class No. 3 of upper protrusions. Instances frequently arise where the position and labial inclination of the upper anterior teeth produces a relative protrusion of the incisal zone and the retrusion of the apical zone. That is, of the cutting edges of teeth and lower part of upper lip and depression of upper portion of upper lip, deepening the naso-labial depression. This condition is beautifully illustrated in model twelve—the labial inclination of the incisors, a depression of the incisive fossa, the roots of teeth being brought forward. If the depression of No. 1 be not too pronounced, it may be restored by slight forward movement of anterior apical zone, with retrusion of incisal zone, with a view of producing a fulcrum at the lingual margins of the alveolus.

Figures 12 and 13 show models of a face of this character before and after treatment. The models of teeth which I here present are marked with the same numbers. The upper first bicuspids had been extracted some time before patient was presented for treatment. The changes brought about in this instance may be diagramatically illustrated with the chart. Chart made by outlining this model. Retrusion of upper portion of upper lip and protrusion of lower portion. The apparatus consisted of appliance for tipping forward the incisal edges of teeth.

Class No. 4 of upper protrusions. There is another quite as common though not so frequently recognized an abnormality—lingual inclination with protusion of apical zone and maxilla, upper portion of upper lip protruding. These patients close their lips with difficulty, and if they laugh we have exposure of upper front teeth and gums. The teeth of this class are commonly irregular in length owing to lingual inclination; the incisal zone may be in proper position. The fact that the roots of cuspids are surrounded by most dense portion of alveolar process, the removal of this makes this operation one of the most difficult in dental orthodontia.

Figures 17 and 18 are from models of a patient over twenty years of age, and will serve to illustrate a case before and after treatment of abnormal protrusion of roots. By retruding upper portion of lip, turning the nose slightly, we have that face. If regulating appliances are properly constructed so that the incisal ends of teeth will not be moved and the entire force of machine may be directed and maintained on the roots, perfect results will follow.

Class No. 5. Another very interesting class that is commonly but wrongfully classed under the head of normal upper protrusions is shown in Figures 19, 20, 21 and 22. In most cases of this class we should have a decided retrusion of the upper anterior teeth. We should extend the arch or extract the bicuspids. In both of these cases the whole operation was performed on the lower. The cases seem to demand a decided retrusion of the upper anterior teeth, in most instances necessary to extract a bicuspid from each side. In a careful study of the profile with a view of determining the æsthetic relations of different segments in the changeable area to the other area, the end of the nose and upper portion of upper lip with that of the chin on Nos. 1 and 4 are in harmonious relation, while the lower No. 2 is slightly protruded, this is due to slight retrusion of lower lip on segment No. 3. We have slight retrusion of incisal portion of upper teeth, the principal portion of operation being on the lower anterior teeth, we protrude them bodily, together with the roots of same. We fill out all of the depression beneath the lower lip allowing to rise slightly, recluding incisal portions of upper teeth; in this we allow the upper lip to drop down.

### UPPER DENTAL AND MAXILLARY RETRUSIONS.

Facial imperfections, which are due to insufficient fullness of contour in the central incisors of the physiognomy are quite common and vary in degree.

Class No. 1. Retruded upper incisors of maxillary process. The physiognomy often appears flattened, with prominent cheek bones and upper lip. Upper incisors appear retruded from the lower incisors. Nos. 1 and 2 represent that particular class of retruded uppers. The upper incisors, which alone have their origin in the inter-maxillary process, are posterior to the normal position. We have deepened incisive fossa. The upper lip resting upon retruded teeth and overlying process is proportionately depressed. The entire lower portion of nose, supported as it is by nasal cartilages, is often decidedly affected in shape by retruding influence of this.

Class No. 2. Retruding dental and maxillary process, due to retrusion of maxillary process. The physiognomy about same. We have less pronounced naso-labial fullness. End of nose may be depressed. A depression of central incisors is often mistaken for protruding jaw. A slight treatment for slight protrusions of lower jaw will produce a protrusion in the facial aspect. Dentists have often tried to retrude the chin. If we could do this we would produce a change rather favorable but would be wrong, for the chin is all right, but in this case the proper treatment is a forcing forward of upper incisive teeth.

